# Modelling constructivist language acquisition through syntactico-semantic pattern finding

**DOI:** 10.1098/rsos.231998

**Published:** 2024-07-24

**Authors:** Jonas Doumen, Katrien Beuls, Paul Van Eecke

**Affiliations:** ^1^ Department of Linguistics, Faculty of Arts, KU Leuven, Leuven, Belgium; ^2^ Itec, Imec research group at KU Leuven, Kortrijk, Belgium; ^3^ Foundations of Computer Sciences, Faculté d’informatique, Université de Namur, Namur, Belgium; ^4^ Artificial Intelligence Laboratory, Vrije Universiteit Brussel, Brussels, Belgium

**Keywords:** language acquisition, computational construction grammar, agent-based modelling

## Abstract

The constructivist acquisition of language by children has been elaborately documented by researchers in psycholinguistics and cognitive science. However, despite the centrality of human-like communication in the field of artificial intelligence, no faithful computational operationalizations of the mechanisms through which children learn language exist to date. In this article, we fill part of this void by introducing a mechanistic model of the constructivist acquisition of language through syntactico-semantic pattern finding. Concretely, we present a methodology for learning grammars based on similarities and differences in the form and meaning of linguistic observations alone. The resulting grammars consist of form-meaning mappings of variable extent and degree of abstraction, called constructions, which facilitate both language comprehension and production. Applying our methodology to the CLEVR benchmark dataset, we provide a proof of concept that demonstrates the online, incremental, data-efficient, transparent and effective learning of item-based construction grammars from utterance–meaning pairs.

## 1. Introduction

Usage-based theories of language acquisition argue that the ability of children to learn language is based on two general cognitive capacities, which are often referred to by the terms *intention reading* and *pattern finding* [1,2]. Intention reading refers to the capacity of children to share attention, recognize gestures and understand the communicative intentions of their interlocutors. It embodies the functional, meaningful dimension of linguistic communication. The second cognitive capacity, pattern finding, refers to the ability of children to recognize similarities and differences in their sensory-motor experiences, and to use this ability for perceptual and conceptual categorization, schema formation, frequency-based distributional reasoning and analogical thinking [1, p. 3−4]. Pattern finding thus provides the mechanisms for generalizing across different communicative interactions, thereby constructing abstract schemata that represent the linguistic knowledge of a language user. In the context of language acquisition, intention reading and pattern finding can be considered two key cognitive capacities that are highly complementary. Intention reading allows a language learner to reconstruct the meaning of an utterance that they observe during a communicative interaction. Pattern finding then provides the mechanisms to learn a grammar based on the combination of these observed utterances and their reconstructed meanings. Such a grammar takes the form of a collection of form-meaning pairings, called *constructions*, which can range from holophrastic mappings between entire utterances and their meaning to abstract mappings between, for example, argument roles and their morpho-syntactic realization patterns [3–5].

There exists an impressive body of theoretical and empirical evidence for both intention reading [6–9] and pattern finding [10–13]. However, no mechanistic models that provide a faithful operationalization of either of these cognitive processes exist to date. In this article, we aim to fill part of this void by presenting a computational operationalization of pattern finding mechanisms that can bootstrap a construction grammar based on a set of semantically annotated utterances alone. As such, we assume that the outcome of the intention reading process is given, hence the availability of the utterances’ semantic representations, but that no pre-existing morpho-syntactic or other grammatical information is available. The resulting grammars consist of form-meaning pairings that are either holophrastic, item-based or lexical. The first constructions that are learned are holophrastic constructions that map between an entire utterance and its meaning representation. Then, item-based constructions and lexical constructions can be learned by generalizing over similarities and differences between novel linguistic observations and previously acquired constructions. Alongside these constructions, a network of emergent grammatical categories is built up, which captures the links between the lexical constructions and the slots of the item-based constructions that they can fill [4,14]. We provide an initial evaluation of our methodology using the CLEVR benchmark dataset [15], illustrating that it allows for fast, incremental and effective learning of constructions and categories. The result of this learning process is a fully operational, productive construction grammar that can be used for both language comprehension and language production, respectively defined as mapping from an utterance to a representation of its meaning and from a meaning representation to an utterance that expresses it [16,17].

Throughout this article, we adopt the terminology and framework proposed by Tomasello [1] for reasons of clarity and consistency. There exist many other approaches to constructivist language acquisition in the fields of developmental psychology and usage-based linguistics, which concretize similar ideas in one form or another [6,9,13,18–29]. A very different perspective is taken by approaches that argue that linguistic structures are to a considerable extent innate [30,31], thereby leaving the usage-based perspective adopted in this article.

The scientific contribution of the research presented in this article is threefold. First of all, it provides computational evidence for the cognitive plausibility of usage-based theories of language acquisition by introducing a mechanistic model of the acquisition of item-based construction grammars and grammatical categories from utterance–meaning pairs. Second, it corroborates the theoretical underpinnings of construction grammar theories, in particular concerning the dynamic and emergent nature of grammatical categories [4,32] and the role these play in facilitating the free combination of constructions [13,33]. Finally, the techniques that we present pave the way for learning computationally tractable, large-scale, usage-based construction grammars that facilitate both language comprehension and production. Apart from their theoretical importance, such grammars are also highly valuable for a large range of application domains, including intelligent conversational agents (e.g. [34,35]), grounded language understanding (e.g. [36,37]), intelligent tutoring (e.g. [38,39]) and the semantic analysis of discourse (e.g. [40,41]).

## 2. Background and related work

### 2.1. Theoretical and empirical foundations

Constructivist theories of language acquisition argue that linguistic structures and categories are not innate, but gradually acquired by children based on the communicative interactions that they take part in [1,14,42–45]. As this process unfolds, the dynamic system of form-meaning pairings and grammatical categories that they have acquired becomes progressively more abstract, organized and efficacious in serving their communicative needs. At an early age, children start to acquire holistic mappings between the linguistic forms that they observe and the meanings that they infer through intention reading. These mappings, which are called *holophrastic constructions*, are holistic in the sense that children do not further decompose them into smaller units. Holophrastic constructions can correspond to single words in adult speech, such as ‘bike’ or ‘juice’, as well as to expressions that are compositional in adult speech but not in early child speech, such as ‘lemme-see’ or ‘I-wanna-do-it’ (examples from [46]).

Somewhat later in their linguistic development, children start to generalize over these holophrastic constructions and acquire *item-based constructions*. This involves a segmentation process in which both the form and the meaning side of a holophrastic construction are (partially) decomposed. In other terms, the child discovers that parts of the form of a holophrastic construction correspond to specific parts of its meaning. For example, the holophrastic constructions ‘eat-apple’ and ‘eat-cookie’ can be generalized into a productive item-based construction ‘eat-X’. This construction captures the relationship between the word form ‘eat’, a possible eating event, and the fact that the referent of X is the patient of this event. This schematization process not only facilitates the generalization of holophrastic constructions to item-based constructions, but also the further generalization of item-based constructions into more abstract constructions that capture for example argument structure relations.

The schematization of more concrete constructions into more abstract constructions, for example, from holophrastic constructions to item-based constructions, involves the creation of slots that can be filled by different possible elements. For example, in the case of the ‘eat-X’ construction, X can be filled by a variety of lexical items, such as ‘banana’, ‘pear’ or even ‘teddy-bear’. However, other lexical items that a child might know, like ‘gone’, ‘green’ or ‘lemme’, are much less likely to fill this slot. Like the constructions themselves, the grammatical knowledge of the association strength between lexical items and grammatical slots is dynamically built up through language use. This grammatical knowledge can be represented in the form of a network that captures the distribution of slots and their fillers. Such a network is in essence a representation of the grammatical categories that underlie the language of an individual. In line with among others [14] and [4], we do not conceive categories as predefined sets to which individual words need to be assigned, but as abstractions over usage patterns that have been observed.

### 2.2. Mechanistic models of usage-based language acquisition

While there exists an overwhelming body of theoretical and empirical studies on child language acquisition, mechanistic models that provide a precise operationalization of the processes through which constructions and categories are acquired are still in their late infancy. Such models are, however, of crucial importance to achieve a full understanding of how children acquire language. They would also provide key evidence against the hypothesis that language cannot be acquired without relying on innate linguistic structures or categories (cf. [47,48]). Moreover, such models would have an important impact beyond the domain of child language acquisition, e.g. concerning the representation of constructions and categories in the field of construction grammar, and the development of intelligent agents in the field of artificial intelligence (e.g. [49]).

Prior mechanistic models that operationalize the learning of constructions can be divided into three groups, based on their definition of the learning task and on the input that they provide to the learning process. A first class of models learns constructions from utterances with their meaning representation. Chang [50] introduces a set of mechanisms for acquiring new constructions based on either input data or previously acquired constructions. Constructions are learned from input data through mapping operations that associate an observed form with its meaning (observation → ‘throw-ball’). Other constructions are not learned from input data but by reasoning over existing constructions. Reorganization operations can recombine structural elements of existing constructions, by merging (‘throw-block’ + ‘throw-ball’ → ‘throw-toy’), joining (‘human-throw’ + ‘throw-bottle’ → ‘human-throw-bottle’) or splitting (‘throw-frisbee’ + ‘throw’ → ‘frisbee’). The learner has an initial grammar consisting of lexical constructions that are associated with pre-established grammatical categories that represent concrete objects, actions and relations. Tellier and Abend *et al*. [51,52] present a Bayesian approach to learning categorial grammars from utterances that were annotated with a semantic representation expressed in first-order logic. Gerasymova & Spranger [53,54] investigate the acquisition of holophrastic constructions, item-based constructions and abstract constructions for Russian aspectual marking in a tutor–learner language game setting [55,56]. Holophrastic constructions are learned by a straightforward mapping operation between an observed form and its meaning. Item-based constructions and abstract constructions are learned as generalizations over pre-categorized lexical items. Beuls *et al*. [57] apply the same methodology to the conjugation of verbs in Hungarian, with a special focus on its intricate agreement marking system. Spranger and Steels [58,59] also apply the same methodology to spatial expressions. Spranger [60] presents an extension to this methodology that learns constructions through more fine-grained semantic-based generalizations. The semantic classes are predefined in an ontology that the learners can access. Dominey and Boucher [61–64] present a neural model for the acquisition of holophrastic constructions, item-based constructions and abstract constructions that capture argument structure relations (e.g. transitives and ditransitives). Learners start with the capability to distinguish between closed-class and open-class words and learn to map between slots in the argument structure constructions and the semantic roles they take. Finally, Van Eecke and Beuls [33,65,66] introduce a set of general operators for learning novel constructions based on the generalization and specialization of existing constructions with respect to novel observations. They apply these operators to an experiment on the emergence of word order in primitive noun phrases and show how a range of concrete to abstract constructions along with a network of emergent grammatical categories can evolve in a population of agents. Nevens *et al*. and Doumen *et al*. [67,68] present evolutions of this experiment that extend its scope to utterances of higher morpho-syntactic and semantic complexity.

A second class of mechanistic models, as introduced by Gaspers *et al*. [69], takes utterances accompanied by a description of the situational context as input. The situational context is represented as a sequence of terms (predicates with their arguments) where one of the terms corresponds to the meaning representation of the utterance. The task for the learner is to learn item-based mappings from patterns occurring in the utterances to predicates with their respective arguments. Gaspers *et al*. [69] introduce a methodology for learning these mappings through probabilistic cross-situational learning and apply this methodology to the Robocup Soccer corpus [70]. A vocabulary is computed first, after which item-based mappings are induced. Except for a segmentation into words, no prior morpho-syntactic or semantic information is provided. Along with the constructions, a network of associations between lexical items and construction slots is built up, corresponding to a system of grammatical categories. Gaspers *et al*. [71–73] present evolutions of this model, where the level of segmentation of the input is reduced from words through graphemes to phonemes. Compared with the first class of models, the difficulty here lies in learning constructions under referential uncertainty, as the exact meaning of the utterances is not provided. On the other hand, the utterances are rather short and always correspond to a single term in the situational context.

A third set of models approach the acquisition of constructions as a traditional unsupervised grammar induction problem. In these models, the goal is not to learn a grammar that can be used for language comprehension or production but to capture the constructions of a language in the form of a grammar with minimal size and maximum coverage. The input to these systems can be parse trees as in the case of [74] or textual material possibly augmented with part-of-speech tags, semantic tags and dependency relations as in the case of [75–77].

The task definition of the first class of models, namely learning constructions based on utterances along with a representation of their meaning, matches our purposes most closely. Whereas the task definition of the second class of models—namely learning constructions under referential uncertainty—is an interesting and important problem, it is beyond the scope of this article. Instead, we will focus exclusively on pattern finding in the sense of [1], and assume that the output of the intention reading process, which deals with the issue of reconstructing the meaning of an utterance, is given.^1^ As a consequence, this allows us to model the acquisition of constructions based on utterances that are morpho-syntactically and semantically of a much higher complexity. The task definition of the third class of models, namely the induction of the syntactic constructions of a language, is less relevant to our goals, as the resulting grammars provide no model of how the induced constructions would interact to perform language comprehension or production tasks.

In general, the existing mechanistic models that learn operational computational construction grammars (i.e. class one and two) have explored interesting ideas on a rather small scale, either because they were limited to specific linguistic phenomena [33,53,54,57–60,65,66], or because of the limited morpho-syntactic and semantic complexity of the input utterances [50,52,61,62,64,69,71–73]. In all of the aforementioned work except [67,68], either a segmentation of the input utterances, a lexicon or a set of predefined grammatical categories was provided. With the exception of [67–69,71–73], the corpora that were used to learn and evaluate the models were not made available and were not described in sufficient detail to make reproduction and comparison feasible.

The methodology that we will present in the next sections pushes the state of the art by introducing a model of how constructions and grammatical categories can be learned from utterance–meaning pairs using general learning operators. We will use a large, semantically annotated corpus that is freely available, with sentences that considerably exceed the morpho-syntactic and semantic complexity of those used in previous work on learning construction grammars from utterance–meaning pairs.

## 3. Data

There are two main requirements for datasets to be compatible with the methodology that we present in this article. First of all, they need to consist of utterances that are annotated with a representation of their meaning. Second, they need to be large enough so that they contain enough utterances that are similar to each other, but not equal, in terms of either form or meaning. The availability of exemplars that are sufficiently close to each other is a necessary precondition for any generalization process and is fully consistent with the prevailing hypotheses of how children acquire language (e.g. [1]). The exact required size of a dataset is as a consequence directly related to the variety and the degree of complexity of the utterances and meaning representations that it contains.

**Figure 1.**
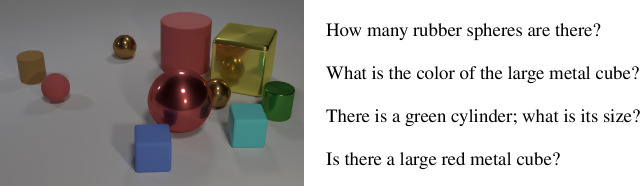
An example scene from the CLEVR dataset with four accompanying questions.

**Figure 2.**
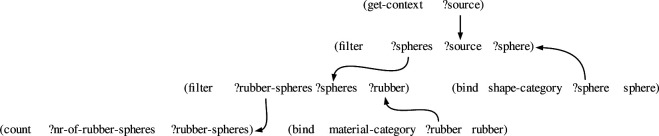
Procedural semantic annotation of the question *How many rubber spheres are there?* Variables are preceded by a question mark.

In this article, we present and evaluate our methodology using the CLEVR dataset [15]. The utterances in the dataset are semantically annotated and the dataset contains ample examples of utterance–meaning pairs that are similar but not equal to each other. The utterances are English questions about images of scenes depicting different configurations of geometrical figures. Each question is annotated with a semantic representation that captures the logical meaning that underlies it. An example of such a scene with a series of accompanying questions is shown in figure 1. The semantic representation of the first utterance, namely *How many rubber spheres are there?*, is shown in figure 2.

The semantic representation in figure 2 takes the form of a set of predicates that share arguments with each other. In the figure, the predicates are drawn in the form of a network, based on the variables that they share. The meaning representation of a question can naturally be represented as a query, i.e. a series of steps that need to be taken in order to answer the question. Each predicate represents a step in this reasoning process, and intuitively corresponds to an atomic cognitive operation that a human or machine can perform. In the case of the example utterance *How many rubber spheres are there?*, the reasoning process consists of four main steps. The first predicate, get-context, binds the image to the variable ‘?source’. Then, the filter predicate filters the image for instantiations of the concept of sphere. The result of this filtering operation, i.e. the set of all spheres that are in the image, is bound to the variable ‘?spheres’. This set of spheres is subsequently filtered by another filter predicate for instantiations of the concept of rubber. The resulting set of rubber spheres is bound to the variable ‘?rubber-spheres’. Finally, the set of rubber spheres is counted by the count predicate and the result is bound to the variable ‘?nr-of-rubber-spheres’. The meaning of the question *How many rubber spheres are there?* corresponds thus informally to filtering an image for spheres, filtering the spheres for rubber objects and counting the result of this last operation. Such meaning representations are called *procedural semantic representations* as the representations themselves are at the same time executable procedures [79–81]. Our methodology handles procedural semantic representations without problems, but is in no way restricted to it. It can handle any semantic representation, as long as it embraces some notion of compositionality and can be expressed as a set of predicates. Examples of other compatible semantic representations include abstract meaning representation [82], PropBank frames [83] and the lambda calculus [84,85].

The CLEVR dataset consists of three splits: a training split of 70 000 images and 699 989 questions, a validation split of 15 000 images and 149 991 questions and a test split of 15 000 images and 149 988 questions. The questions in the training and validation splits come with semantic annotations, whereas the test set does not. As we require these annotations in order to evaluate our model, we use the training split of the CLEVR dataset as training set and the validation split as the test set. The question–annotation pairs embrace various aspects of reasoning, including attribute identification (*There is a large cube; what is its colour?*), counting (*How many green spheres are there?*), comparison (*Are there an equal number of large cubes and small things?*), spatial relationships (*What size is the cylinder that is right of the yellow shiny thing that is left of the cube?*) and logical operations (*How many objects are either red cubes or yellow cylinders?*). For the purposes of this paper, we have selected the subset of CLEVR questions that do not involve comparison, spatial relationships or logical operations. The main reason for this is that these are complex cognitive operations that often correspond to long and complex utterances that are far removed from the linguistic expressions that children (or even other humans) are faced with. Our final training and test sets consist of 47 134 questions and 10 044 questions, respectively. For illustrative purposes, a sample of the questions is included in appendix A.

## 4. Methodology

We will first present the intuition behind our novel methodology for learning constructions from observations (§4.1) and then discuss a computational operationalization of this methodology (§4.2).

### 4.1. Learning constructions through generalization

The input to the learning process are utterances that are annotated with a representation of their meaning. The output of the learning process should consist of form-meaning mappings (constructions) that can be used for comprehending and producing utterances. Some degree of generality is necessary, as the learned constructions should not only be able to process previously encountered utterances, but also be able to handle novel ones.

Let us for a moment take the perspective of the learning algorithm. At the beginning of the learning process, the construction inventory is empty and the first utterance–meaning pair from the corpus comes in. At this point, the only thing that the learning algorithm can do is to store an exact mapping between the observed form and its meaning. Such a holistic mapping corresponds to a holophrastic construction and is usable as such, albeit only for comprehending and producing the exact same utterance as the one that was observed. In order to use such a construction in the comprehension direction, it suffices to match the form side of the construction with an utterance and return the meaning side of the construction if the matching process succeeded. In order to use the same construction in the production direction, the meaning side of the construction must be matched with a semantic network and the form side must be returned.

When a next observation comes in, the learning algorithm first checks whether it is already covered by constructions that have been acquired previously. When this is the case, the constructions that are involved in the successful comprehension and production of the observation are reinforced by incrementing their entrenchment score. If the observation is not covered, the algorithm checks whether there are any generalizations that can be made based on the combination of the novel observation and any previously acquired constructions. It is these generalization mechanisms that embody the pattern finding capacity and are thereby at the core of the construction learning process. We have identified three classes of mechanisms that facilitate the learning of general constructions by algorithmic reasoning over similarities and differences between existing constructions and novel observations.

#### 4.1.1. Generalizing over holophrastic constructions

The first class of mechanisms facilitates the generalization of holophrastic constructions with respect to novel observations. These mechanisms can learn item-based constructions that capture the similarities between a novel observation and an existing holophrastic construction that was learned based on a similar, but not equal, observation. These item-based constructions abstract away from the differences between the observation and the holophrastic construction.

For example, imagine that a holophrastic construction has already been learned based on the observation of the utterance *How many rubber spheres are there?* and the semantic network shown in figure 2. Now, a novel utterance *How many rubber cubes are there?* is observed, along with a very similar meaning network in which the predicate ‘(bind shape-category ?cube cube)’ appears at the place of ‘(bind shape-category ?sphere sphere)’. The generalization mechanisms compute the similarities and differences between the construction and the observation in terms of both form and meaning, and make a new item-based construction that maps between the utterance *How many rubber ?X are there*? and the semantic network from figure 2 in which the non-overlapping predicate has been omitted. At the same time, two new lexical constructions are created, which capture the differences between the observation and the original holophrastic construction. In our example, these will be a construction that maps between the utterance *cubes* and the meaning representation ‘(bind shape-category ?cube cube)’ and a construction that maps between the utterance *spheres* and the meaning representation ‘(bind shape-category ?sphere sphere)’. Finally, categorial links are made between the ?X slot in the item-based construction and the new lexical constructions. The learning algorithm reveals here that *cubes* and *spheres* can appear in the same slot of the item-based construction and that they are therefore close to each other in terms of grammatical categories. Note that the ontological classes that appear in the meaning predicates (e.g. shape-category and colour-category) are not used at all by the learning algorithm, and that the grammatical categories emerge solely from associations between slots and their observed fillers. The ontological categories are kept because they are part of the procedural semantic annotation, but could be omitted without any consequences. The term ‘lexical construction’ is used to refer to a construction that has been learned as a slot-filler in an item-based construction, as is common in the language acquisition literature. This is for clarity reasons only. Following the construction grammar tradition, the learning algorithm does not have any notion of ‘word’ or ‘lexeme’ and does not formally distinguish between different types of constructions.

There are three different scenarios in which mechanisms of this class are active. The first scenario concerns utterances that extend holophrases that are already known. An example would be the generalization of *Are there any cylinders?* to *Are there any red cylinders?* In this case, an item-based construction *Are there any ?X cylinders?* is learned, along with a lexical construction for *red* and a categorial link between the lexical construction and the open slot in the item-based construction. The second scenario concerns utterances that reduce known holophrases. An example would be the reduction of *What is the size of the metal block?* to *What is the size of the block?*. In this case, an item-based construction for *What is the size of the ?X block?* is learned, along with a holophrastic construction for *What is the size of the block?*, a lexical construction for *metal*, and a categorial link between the slot in the item-based construction and the lexical construction. The final scenario concerns utterances that are not a mere extension or reduction of each other, but contain different formal and/or semantic material. An example would be the utterances *How many rubber spheres are there?* and *How many rubber cubes are there?* discussed above, where a holophrastic construction for *How many rubber spheres are there?* is already in place. An item-based construction for *How many rubber ?X are there* is learnt along with a lexical construction for *cubes* and a categorial link between the open slot in the item-based construction and the new lexical construction. Additionally, a second lexical construction for *spheres* is learned, along with a categorial link between the open slot in the item-based construction and the lexical construction for *spheres*.

#### 4.1.2. Learning constructions based on a partial analysis

The second class of mechanisms is designed to handle cases where an observation could not completely be processed using the existing constructions of grammar, but where a partial analysis could be provided. These mechanisms can then create novel constructions that can work together with existing constructions so that the entire observation can be processed successfully. They start thus from the combination of a novel observation on the one hand, and an item-based construction or one or more lexical constructions on the other. The second class of mechanisms is active in two different scenarios.

The first scenario concerns observations to which an item-based construction can apply, but where there remains material that is not covered by any of the existing constructions. An example would be an observation of *What is the size of the green block?*, where a construction for *What is the size of the ?X block?* is already known, while no construction for *green* has been learned yet. The learning algorithm detects that some aspects of the form and the meaning of the observation are not covered by the existing item-based construction and it creates a novel lexical construction that maps between those parts of the form and meaning that were not covered. Additionally, a categorial link is made between the slot in the item-based construction and the lexical construction. In our example, this means that a lexical construction for *green* is learned, along with a categorial link between this construction and the ?X slot in the construction for *What is the size of the ?X block?*

The second scenario concerns observations to which one or more lexical constructions can apply, but where these constructions do not fully cover the input. An example would be an observation of the utterance *There is a big red cube; what is its material?*, where lexical constructions for *big*, *red*, *cube* and *material* have already been learned. The learning algorithm will then create a new item-based construction that incorporates all the form and meaning material that remains after the application of these lexical constructions, and that abstracts away from these constructions through the integration of four slots. The result is an item-based construction of the form *There is a ?A ?B ?C; what is its ?D?* and four categorial links from the existing lexical constructions to the slots in the new item-based construction.

#### 4.1.3. Extending the categorial network

The third class of mechanisms is designed to handle cases where all necessary constructions are already in place, but where they cannot combine owing to the absence of certain links in the categorial network. An example would be the utterance *How many things are there?* where an item-based construction covering *How many ?X are there?* and a lexical construction covering *things* already exist, but where there is no link in the categorial network between the lexical construction for *things* and the ‘?X’ slot in the item-based construction. In such cases, the learning algorithm adds the missing link to the categorial network.

### 4.2. Computational operationalization

While §4.1 has provided a high-level introduction to the intuition behind the methodology that we have developed, the present section presents a full computational operationalization. It starts by introducing representations and processing mechanisms for constructions (§4.2.1), as well as a meta-level architecture for problem-solving and learning (§4.2.2). It then provides a more detailed description of an operationalization of the construction learning mechanisms that were introduced above, and highlights their integration into the learning architecture (§§4.2.3 to 4.2.8).

#### 4.2.1. Representing linguistic knowledge as constructions

Constructionist approaches to language, as pioneered by among others [3,4,10,86], argue that the linguistic knowledge of a language user can be captured in the form of a collection of learned form-meaning mappings, called constructions. Constructions can map between form and meaning patterns of any extent and degree of abstraction, ranging from individual morphemes (e.g. dog-cxn) and idiomatic expressions (e.g. break-a-leg-cxn), over partially instantiated structures (e.g. x-take-y-for-granted-cxn) to fully abstract schemata (e.g. the resultative or ditransitive construction). During language processing, the constructions of grammar freely combine in order to comprehend or produce linguistic expressions [13].

The field of computational construction grammar computationally operationalizes the basic tenets of construction grammar so that constructions can be represented formally and processed algorithmically [33]. For the purposes of this study, we represent and process the constructions that are learned using the Fluid Construction Grammar framework (FCG—[16,17,87,88]). FCG is in essence a special-purpose programming language that provides adequate abstractions and useful building blocks for implementing computational models of constructional language processing.

**Figure 3.**
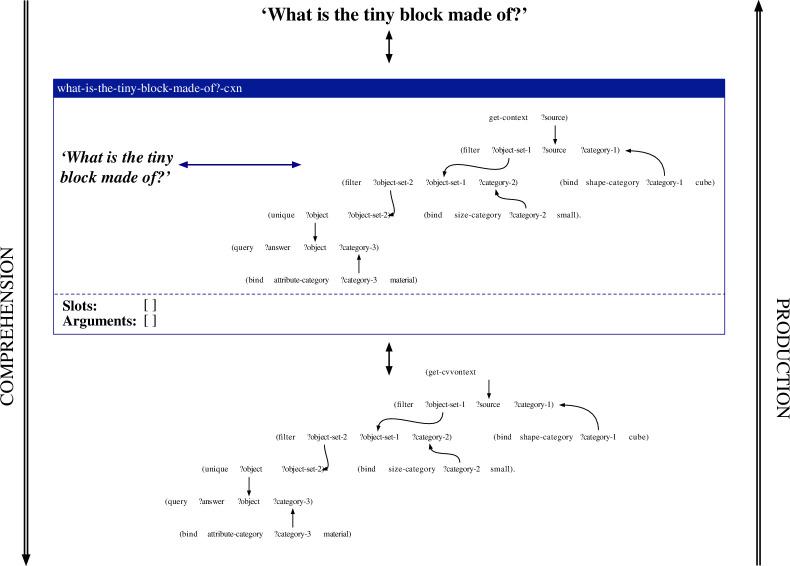
Schematic representation of the application of the holophrastic construction what-is-the-tiny-block-made-of?-cxn in the comprehension and production directions. The form pole of the construction is shown on the left side of the double arrow, while its meaning pole is shown on its right side. In the comprehension direction, shown from top to bottom, the form pole of the construction is matched with the input utterance, and its meaning pole is returned. In the production direction, shown from bottom to top, the meaning pole of the construction is matched with the input meaning representation and its form pole is returned.

Constructions are represented as pairings between form and meaning. Both the form and the meaning can be of arbitrary complexity and degree of abstraction. The most straightforward case concerns a holophrastic construction, which captures a direct mapping between a complete utterance and a representation of its meaning. An example of such a construction is shown in the middle of figure 3. This construction captures the pairing between the utterance *What is the tiny block made of?* (left of the double arrow) and a procedural semantic representation that filters a scene for cubes, filters the result for small things, checks whether there is only one resulting object, and queries its material (right of the double arrow). During language processing in the comprehension direction, flowing from top to bottom in the figure, the construction matches its form pole with the observed utterance (shown at the top) and returns its meaning pole if matching succeeded (shown at the bottom). In the production direction, flowing from bottom to top in the figure, the construction matches its meaning pole with the input meaning representation (shown at the bottom) and returns its form pole (shown at the top). As visualized through the empty ‘slots’ and ‘arguments’ lists in the construction, holophrastic constructions do not contain any slots that can be filled by other constructions and do not provide any arguments that could fill open slots in other constructions.

**Figure 4.**
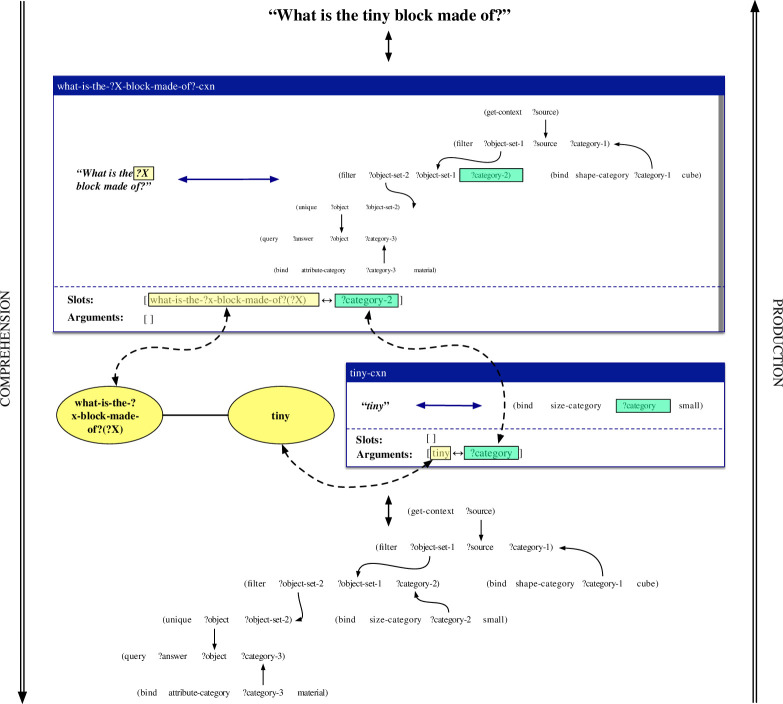
Schematic representation of how the item-based construction what-is-the-?x-block-made-of?-cxn and the lexical construction tiny-cxn collaboratively process the utterance *What is the tiny block made of?* in the comprehension and production directions. The tiny-cxn thereby fills the ‘?X’ slot in the what-is-the-?x-block-made-of?-cxn.

A case in which an item-based construction and a lexical construction collaboratively process the same utterance as above is exemplified in figure 4. The item-based construction what-is-the-?x-block-made-of?-cxn maps between the pattern *What is the ?X block made of?* and a procedural semantic representation that filters the scene for cubes, filters the result for a variable property, checks whether there is only one resulting object and queries the material of this object. The construction thus contains an open slot on both its form pole and its meaning pole. The open slot on its form pole concerns a variable element to be positioned between *What is the* and *block made of?* and is highlighted in yellow in the figure. The open slot on its meaning pole concerns the unbound variable ‘?category-2’, representing the concept to be used by the second filter operation, and is highlighted in green. The coupling between both open slots is specified in the slot specification of the construction. This coupling states that the slots can be filled by some other construction that provides a mapping between something of category ‘what-is-the-?x-block-made-of?(?X)’, representing the ‘?X’ slot in the what-is-the-?x-block-made-of?-cxn, and something that matches the variable ‘?category-2’. The lexical construction tiny-cxn maps between the string *tiny* and the meaning representation ‘(bind size-category?category small)’. This construction contains no open slots itself, but provides arguments that can fill open slots in other constructions. In this case, it can fill the open slots in the what-is-the-?x-block-made-of?-cxn as the category ‘tiny’ is connected to the category ‘what-is-the-?x-block-made-of?(?X)’ through the categorial network. At the same time, the ‘?category’ variable from the tiny-cxn can unify with the ‘?category-2’ variable from the what-is-the-?x-block-made-of?-cxn. The result is the utterance *What is the tiny block made of?* in the production direction, and a complete semantic network for this utterance in the comprehension direction.

If multiple combinations of constructions can apply to the same input utterance or meaning representation, the combination with the highest average entrenchment score will be preferred. We refer the interested reader to [89] for a detailed discussion of how constructional language processing is concretely operationalized in FCG.

#### 4.2.2. Routine processing versus meta-level processing

Following insights from the fields of artificial intelligence [90,91], cognitive science [92] and neurolinguistics [93,94], we distinguish between two levels of processing: routine processing and meta-level processing. Routine processing deals with comprehending and producing utterances that are covered by the existing constructions of grammar. During routine processing, a meta-level architecture is constantly monitoring the application of constructions and diagnoses any problems that might occur. If a problem occurs, it triggers a jump to the meta-level, where repair strategies search for a fix that remedies the problem. When a fix is found, routine processing resumes. If it is the case that the fix ultimately leads to a valid solution, it is consolidated so that it can later be reused during routine processing. In our learning methodology, diagnostics trigger a jump to the meta-level if the grammar cannot provide a correct mapping for a given utterance–meaning pair. Repairs have the task of coming up with fixes that take the form of novel constructions, or nodes and links in the categorial network. These fixes can be consolidated by adding them to the construction inventory or the categorial network of the learner. The use of a meta-level architecture has the advantage that there exists an effective separation between routine processing on the one hand, and problem solving and learning on the other. The idea is that routine processing can be implemented efficiently and that less efficient problem-solving strategies will only become active if an actual problem has been diagnosed. It also allows abandoning the strict distinction between the training phase and the operational phase. As long as some form of feedback is provided, the system can keep learning and adapting also after it has been deployed, which is a highly desirable property of intelligent systems. Meta-level architectures for computational construction grammar have been pioneered by [65,95,96].

#### 4.2.3. Repairs for acquiring holophrastic constructions

When the existing constructions of a grammar are not sufficient to collaboratively map from an input utterance to its meaning representation as annotated as a gold standard in the dataset, the most basic repair strategy consists of creating a new holophrastic construction that captures a mapping between the exact form and its annotated meaning. Holophrastic constructions contain no abstract slots and provide no arguments that can fill slots in other constructions. They can be used in both the comprehension and production direction, but can only apply in cases where the exact same utterance needs to be comprehended or the exact same meaning representation expressed. An example of a holophrastic construction is shown in the middle of figure 3 above. In this example, the what-is-the-tiny-block-made-of?-cxn has been learned based on the observation of the utterance *What is the tiny block made of?* and a procedural semantic representation that filters the scene for cubes, filters the result for small things, checks whether there is only one resulting object, and queries the material of this object.

We will refer to this repair strategy by the name *nothing *→ *holophrase*. While this repair does not make any powerful generalizations, it has the advantage that it always succeeds, which is particularly useful in the earliest phases of the learning process. The resulting holophrastic constructions serve as a basis for the generalization processes implemented by other repair strategies.

#### 4.2.4. Repairs for generalizing over holophrastic constructions

This class of repair strategies facilitates the generalization of holophrastic constructions to item-based and lexical constructions. It handles cases that are not covered by the existing constructions of a grammar, but where a holophrastic construction already exists that covers an utterance–meaning pair that is similar, but not equal to an observation. Our implementation includes three repairs of this class.

**Figure 5.**
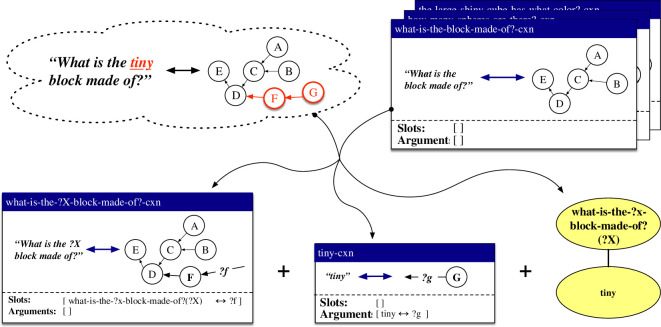
Based on a new utterance–meaning pair (upper-left corner) and an existing holophrastic construction (upper-right corner), the repair strategy *holophrase *→ *item-based + lexical – addition* learns an item-based construction (bottom-left corner) and a lexical construction (bottom centre), along with a link in the categorial network (bottom-right corner).

The first repair handles cases where an observation is an extension of the form-meaning mapping captured by a holophrastic construction. The extension can be on the form side, on the meaning side or on both sides. An instantiation of this repair is shown in figure 5. The observation, shown in the upper left corner, concerns the utterance *What is tiny block made of?* (left of the double arrow) along with a procedural semantic network, represented here in an abstract fashion for space reasons (right of the double arrow). This observation extends the existing holophrastic construction what-is-the-block-made-of?-cxn, shown in the upper right corner, on both the form and the meaning side. Based on the combination of the observation and the holophrastic construction, an item-based construction what-is-the-?x-block-made-of?-cxn and a lexical construction tiny-cxn are created (shown on the bottom of the figure). The item-based construction contains one open slot, as indicated in its slot specification, which can be filled by something that is of type ‘what-is-the-?x-block-made-of?(?X)’ and binds the ‘?f’ variable in its meaning representation. The lexical construction provides an argument of type ‘tiny’ which binds the ‘?g’ variable in its meaning representation, as shown in its argument specification. A link between the categories ‘what-is-the-?x-block-made-of?(?X)’ and ‘tiny’ is also added to the categorial network, as shown in the bottom right corner of the figure. When applying both the item-based construction and the lexical construction, the categories of the argument and the slot match through the categorial network and the open variables in their respective meaning representations are unified. The result is the utterance *What is the tiny block made of?* in the production direction and a fully integrated semantic network in the comprehension direction. We refer to this repair strategy by the name *holophrase *→ *item-based + lexical – addition*.

**Figure 6.**
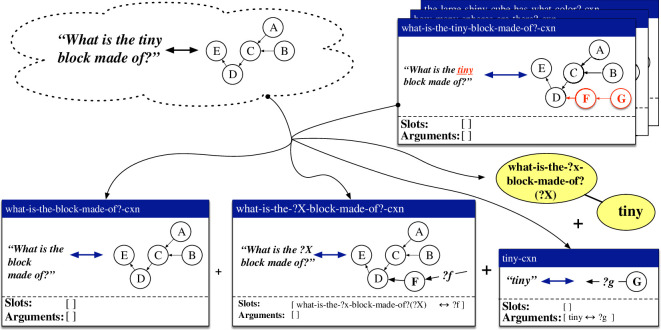
Based on a new observation and an existing holophrastic construction, the repair strategy *holophrase *→ *item-based + lexical + holophrase – deletion* learns a holophrastic construction, an item-based construction and a lexical construction, along with a link in the categorial network.

The second repair, called *holophrase *→ *item-based + lexical + holophrase – deletion*, deals with novel observations that reduce the form and/or meaning of an existing holophrastic construction. An instantiation of this repair is shown in figure 6. In the example, the observation concerns the form and meaning of the utterance *What is the block made of?*, where a holophrastic construction what-is-the-tiny-block-made-of?-cxn already exists. The repair does not only create a novel holophrastic construction what-is-the-block-made-of?-cxn, which covers the observation, but also creates an item-based construction what-is-the-?X-block-made-of?-cxn and a lexical construction tiny-cxn that generalize over the original holophrastic construction. The item-based construction and lexical construction are linked through the categorial network. These constructions are not used to process the current observation, but concern generalizations that could be made based on this observation and a previously acquired holophrastic construction.

**Figure 7.**
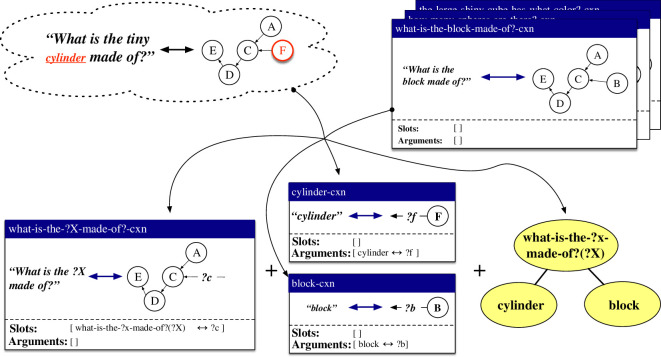
Based on a new observation and an existing holophrastic construction, the repair strategy *holophrase *→ *item-based + lexical + lexical – substitution* learns an item-based construction and two lexical constructions, along with two links in the categorial network.

The third repair, called *holophrase *→ *item-based + lexical + lexical – substitution*, handles cases where both the form and the meaning of an observation differ from an existing holophrastic construction in some aspect. An instantiation of this repair is shown in figure 7. The form and meaning of the utterance *What is the cylinder made of?* are observed here, and a holophrastic construction covering *What is the block made of?* already exists. The repair creates an item-based construction what-is-the-?x-made-of?-cxn along with the lexical constructions cylinder-cxn and block-cxn. Two categorial links are also created, which associate the arguments of the lexical constructions with the open slot in the item-based construction.

During learning, there will often be multiple previously acquired holophrastic constructions that can be used as a basis for generalization. Constructions that share more form or meaning predicates with the observed form-meaning pair are always preferred. If multiple constructions are equally similar to the observation, the construction with the highest entrenchment score (see §4.2.8) is preferred.

#### 4.2.5. Repairs for acquiring constructions based on a partial analysis

A next class of repair strategies handle cases where the existing constructions of a grammar do not fully cover an observation, but where a partial analysis is available. This partial analysis is either the result of the application of one or more lexical constructions, or of the application of an item-based construction, potentially in combination with one or more lexical constructions. These two cases are handled by two designated repairs.

**Figure 8.**
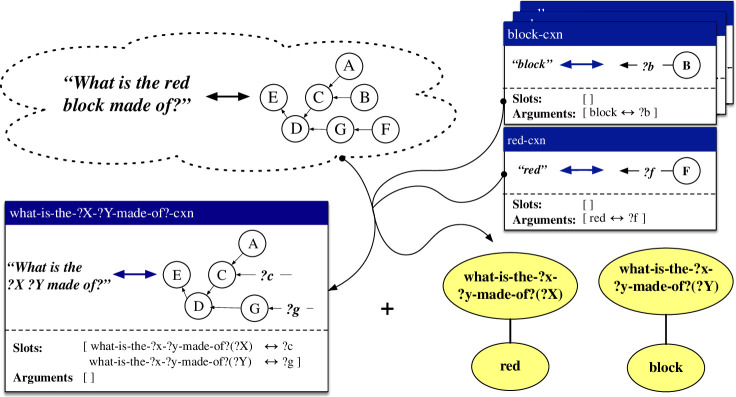
Based on a new observation and one or more existing lexical constructions, the repair strategy *lexical *→ *item-based* learns an item-based construction, along with categorial links between the arguments of the lexical constructions and the open slots in the item-based construction.

The first repair, which deals with cases where one or more lexical constructions could apply, is referred to as the *lexical *→ *item-based* strategy. An instantiation of this repair is shown in figure 8. Here, the observation consists of the form and meaning of the utterance *What is the red block made of?*. Two lexical constructions covering *block* and *red* are already available. The repair builds a new item-based construction what-is-the-?x-?y-made-of?-cxn, which includes two slots. Along with this construction, two categorial links are also learned. The first link captures the association between the red-cxn and the ‘?X’ slot in the item-based construction, while the second link captures the association between the block-cxn and the ‘?Y’ slot in the item-based construction.

**Figure 9.**
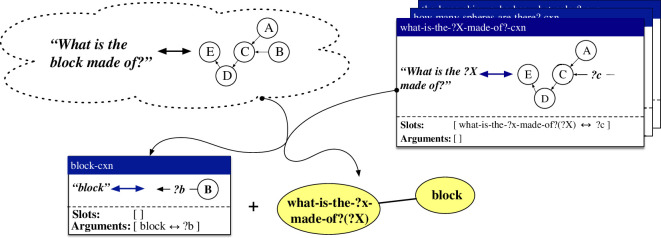
Based on a new observation and an existing item-based construction, the repair strategy *item-based *→ *lexical* learns a lexical construction, along with a categorial link between the argument of the lexical construction and the open slot in the item-based construction.

The second repair, which deals with cases where an item-based construction could apply, is referred to as the *item-based *→ *lexical* repair. An instantiation of this repair is shown in figure 9. Here, the what-is-the-?x-made-of-cxn can apply to an observation of the utterance *What is the block made of?* However, as the grammar does not yet include a construction that covers *block*, the observation cannot be handled successfully during routine processing. The repair creates a new lexical construction, in this case the block-cxn, covering the part of the observation that was not yet covered, as well as a categorial link between the new lexical construction and the respective slot in the existing item-based construction. This repair also handles cases where the partial analysis is the result of the application of an item-based construction in combination with one or more existing lexical constructions.

#### 4.2.6. Repairs for extending the categorial network

The final class of repairs handles cases where all item-based and lexical constructions that are needed to cover an observation are already part of the grammar, but where the categorial network does not yet contain all required links between slots and their fillers.

**Figure 10.**
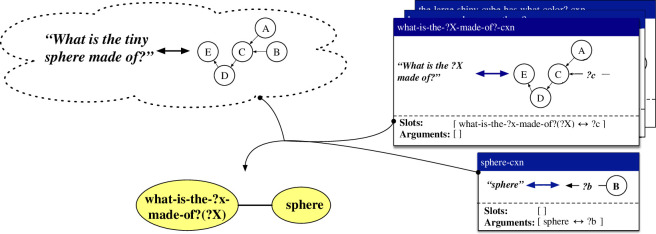
Based on a new observation and a number of existing item-based and lexical constructions, the repair strategy *add-categorial-links* learns one or more categorial links between the lexical constructions and the item-based construction.

This class of repairs has only a single member, namely the *add-categorial-links* repair. An instantiation of this repair is shown in figure 10. The observation concerns an utterance *What is the sphere made of?* along with a representation of its meaning. The item-based construction capturing the pattern *What is the ?X made of?* and the lexical construction covering *sphere* are already part of the grammar, but cannot combine because the categorial network does not contain a link between the argument provided by the lexical construction and the open slot in the item-based construction. The repair detects that it is the absence of a categorial link that blocks the construction application and adds this link to the categorial network.

Note that this repair strategy is somewhat different than the other repair strategies, as its problem-solving capacities do not rely on the gold standard annotation. Through a search process, it can detect the absence of required links in the categorial network when processing an utterance in the comprehension direction or a meaning representation in the production direction.

#### 4.2.7. Competition between repair strategies

Repair strategies are often in competition with each other, as different generalizations can be made based on the same observation and previously acquired constructions. For example, the *nothing *→ *holophrase* repair can always apply, but other repairs lead in many cases to more useful generalizations. We therefore specify the order in which repairs are activated by the meta-level architecture. Only if an earlier repair detects that it cannot adequately handle a problem, does the next repair become active. The order of repair strategies in the meta-level architecture is the following:

- *add-categorial-links*: Problems are preferably solved by adding links between existing nodes in the categorial network, without creating any new constructions.
- *item-based *→ *lexical*: If a lexical construction is missing, it is created along with a link in the categorial network.
- *holophrase item-based + lexical + lexical – substitution*: An item-based and two lexical constructions are created based on an existing holophrastic construction, along with two categorial links.
- *holophrase *→ *item-based + lexical – addition*: An item-based and a lexical construction are created based on an existing holophrastic construction, along with a categorial link.
- *holophrase *→ *item-based + lexical + holophrase – deletion*: A holophrastic construction, an item-based construction and a lexical construction are created based on an existing holophrastic construction, along with a categorial link.
- *lexical *→ *item-based*: An item-based construction with open slots is created, along with categorial links between the arguments of the lexical constructions and these open slots.
- *nothing *→ *holophrase*: If all other repairs fail, a new holophrastic construction is created.

The order of activation of repair strategies reflects that the creation of new categorial links is preferred over the creation of new constructions (repair (i)). If new constructions need to be created, lexical constructions are preferred (repair (ii)). If the creation of lexical constructions does not suffice, the creation of item-based constructions (repairs (iii)–(vi)) is preferred over the creation of new holophrastic constructions (repair (vii)).

#### 4.2.8.Updating entrenchment scores

Psychological and linguistic research has shown that the entrenchment of constructions and categories plays an important role in shaping the grammar of language users [20,97–99]. Entrenchment refers to the process in which grammatical knowledge that is successfully used during communicative interactions becomes more and more engrained over time. Different factors influence the degree of entrenchment of constructions and categories, communicative success and frequency being the most important.

In our model, entrenchment is operationalized through the assignment of scores to constructions and links in the categorial network. During language processing, constructions with higher entrenchment scores are preferred over constructions with lower scores. The entrenchment scores of constructions range between 0 and 1, with 0 indicating minimal entrenchment and 1 maximal entrenchment. When a new construction is created, it is assigned an initial entrenchment score of 0.5. When a construction is successfully used to process an observation from the corpus, its score is increased by 0.1. At the same time, competing constructions are punished by decreasing their scores by 0.3. Competitors are defined as constructions that could also have contributed to the successful analysis of the same observation. There is thus no built-in bias towards more general constructions. However, the fact that more general constructions are applicable in a broader range of situations and are therefore more frequently used, will, owing to the dynamics of rewarding successful usage and punishing competitors, lead to higher entrenchment scores for more general constructions. The exact values by which constructions are rewarded and punished do not influence the global dynamics of the learning process, as long as they are positive and negative, respectively (see earlier work by, among others, [100,101]).

An entrenchment score is also assigned to each link in the categorial network. This score reflects the number of times that a link has been used in language comprehension or production. The entrenchment of the categorial links reveals the association strength between slots and their fillers. Nodes in the network of which the links have a similar distribution can be seen as close to each other in terms of grammatical category. Categorial distance can be computed based on the network using similarity metrics such as (weighted) cosine similarity. While categorial distance is not exploited in the experiments reported on in this paper, it could play an important role in experiments that operationalize (creative) language production strategies.

## 5. Experiments

This section presents a validation of our methodology for acquiring constructions on the CLEVR dataset discussed in §3. We first describe the experimental set-up (§5.1) and then present the evaluation results (§5.2).

### 5.1. Experimental set-up

The primary experiment consists of processing the 47 134 observations from our training set using the meta-level architecture introduced above. For each experimental run, the observations are shuffled, so that any side-effects that might be caused by the order in which the observations are presented are levelled out. The learning operations, which are implemented by means of repair strategies, are only active when an observation cannot be processed successfully by the constructions that have been learned so far. Entrenchment scores are updated after each communicative interaction, also when the activation of the meta-level was not required. Thanks to this experimental design, the learning process by which linguistic knowledge is gradually built up can be monitored in detail. The learning process is evaluated through four quantitative metrics: *communicative success*, *grammar size*, *number of constructions per type* and *active repair strategies*.

— *Communicative success* over time is computed by comparing the analysis of the learner after each observation with the gold standard annotation in the corpus. All observations that could be successfully handled by the routine layer are assigned the value 1. All observations that required the activation of a repair that needs access to the gold standard are assigned the value 0. The remaining observations, which were handled by the repair *add-categorial-links*, are also assigned the value 1. This is because the meta-reflective problem-solving capacity that was needed did not require any external information or feedback. The choice for handling such cases at the meta-level is motivated by reasons of efficiency and conceptual clarity (see §4.2.2). The binary values returned by the communicative success metric are plotted using a sliding window of 50 observations.
— *Grammar size* over time is computed by counting after each observation the number of constructions in the grammar that enjoy at least some level of entrenchment. Constructions in which the entrenchment score has reached 0, corresponding to minimal entrenchment, are not counted.
— *Number of constructions per type* over time counts the constructions in the grammar in the same way as the grammar size metric. However, the constructions are now divided into three groups: holophrastic constructions, item-based constructions and lexical constructions. Constructions with no slots or arguments are counted as holophrastic constructions, constructions with slots and no arguments are counted as item-based constructions, and constructions with arguments and no slots as lexical constructions.
— *Active repair strategies* over time are computed for each repair individually by recording after each observation whether the repair has been active. Depending on whether it was active, a 0 or 1 is recorded. The binary values returned by this metric are plotted using a sliding window of 50 interactions.

A secondary experiment concerns the processing of the 10 044 observations from our test set using the grammar that was learned on the training set. In this experimental set-up, the model has no access to the gold standard and can thus not use any of the repairs except for the *add-categorial-links* repair. While this experiment corresponds exactly to the final phase of the primary experiment, it is reported on in this paper so that later comparison with alternative methodologies will be straightforward. Here, communicative success is averaged over the whole test set. Grammar size and number of constructions per type do not change during the experiment. The active repair strategies metric is not applicable in this experimental set-up.

The experimental results reported below are based on 10 independent experimental runs. The observations were shuffled before each run. The error bars that are plotted represent percentiles 5 and 95.

### 5.2. Results

**Figure 11.**
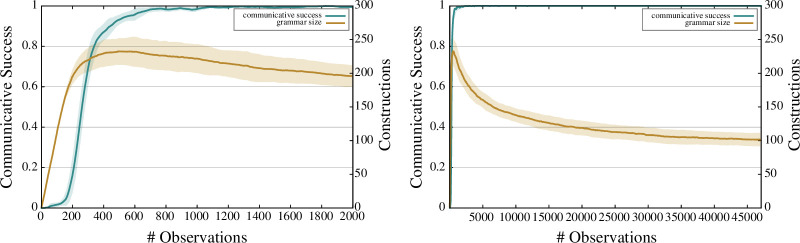
Evolution of communicative success (left *y*-axis) and grammar size (right *y*-axis) over time. The left figure zooms in on the first 2000 observations while the right figure shows a global view of the learning dynamics over the whole dataset. Communicative success is drawn using a sliding window of 50 observations. The error bars correspond to the percentiles 5 and 95 averaged over 10 experimental runs.

**Figure 12.**
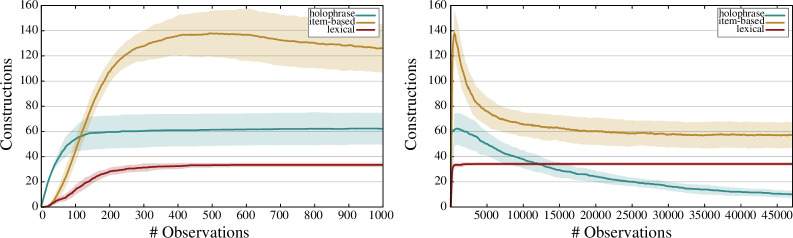
Evolution over time of the number of constructions per type with an entrenchment score > 0. The left figure zooms in on the first 1000 observations while the right figure shows a global view of the dynamics over the whole dataset. The error bars correspond to the percentiles 5 and 95 averaged over 10 experimental runs.

**Figure 13.**
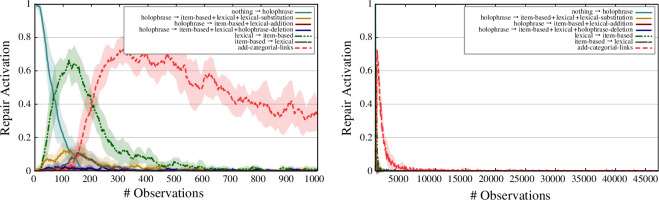
Active repairs over time. The left figure zooms in on the first 1000 observations while the right figure shows a global view of the dynamics over the whole dataset. A sliding window of 50 observations was used. The error bars correspond to the percentiles 5 and 95 averaged over 10 experimental runs.

The results obtained through the primary experiment are shown in figures 11–13. Figure 11 displays the communicative success and grammar size metrics, respectively, on the left and right *y*-axis as a function of the number of observations (*x*-axis). The left graph zooms in on the first 2000 observations while the right graph includes all 47 134 observations from the dataset. We can see that the communicative success starts at 0, as the experiment starts with an empty inventory of constructions. The degree of communicative success rises rapidly, with more than 90% of the observations being successfully processed by the learned grammar after only 500 observations have been encountered. After 2000 observations, communicative success is already achieved in 99.6% of new observations.

The grammar size starts at 0 constructions and grows rapidly in the first phase of the experiment. After 500 observations, the grammar has reached its peak size of around 230 constructions that have some degree of entrenchment. This number then declines as a result of the rewarding and punishing of constructions. At the end of the learning process, the resulting grammar consists of 101.5 constructions on average.

An analysis of the types of constructions that are part of the learned construction inventory is provided in figure 12. The left graph zooms in on the first 1000 observations, while the right graph provides a complete picture. The results show that holophrastic constructions flourish in the earliest phase of the experiment. In a second phase, item-based and lexical constructions take over the role of the holophrastic constructions, with an abundance of item-based constructions being created. Over the course of the experiment, the linguistic inventory of the learner gradually reaches a stable state consisting of a limited number of entrenched lexical constructions and (more general) item-based constructions. At the end of the experiment, the grammar consists on average of 10.2 holophrastic constructions, 57.1 item-based constructions and 34.2 lexical constructions. These results show that the holophrastic constructions have not yet completely disappeared after 47 134 observations and that the theoretical maximum of 35 lexical constructions was attained in 7 out of 10 experimental runs. Note that it is the dynamic evolution of the number of constructions per type over time that is important, rather than the absolute number of constructions at a given moment in time.

Figure 13 shows the active repair strategies over time. The graph on the left zooms in on the first 1000 observations, while the graph on the right captures the full experiment. The graphs show that the *nothing *→ *holophrase* repair is the most active repair in the first phase of the experiment. After that, the *holophrase *→ *item-based + lexical + lexical – substitution* repair, and to a lesser extent the *holophrase *→ *item-based + lexical – addition* and *holophrase *→ *item-based + lexical + holophrase – deletion* repairs are active. As a result of the bootstrapping of item-based and lexical constructions by these repairs, the *lexical *→ *item-based* and *item-based *→ *lexical* repairs become active, with the *lexical *→ *item-based* repair being the predominant repair in this phase of the experiment. Once the inventory of item-based and lexical constructions is in place, the *add-categorial-links* repair can solve almost all remaining problems without relying on the gold standard or any other form of external information.

**Figure 14.**
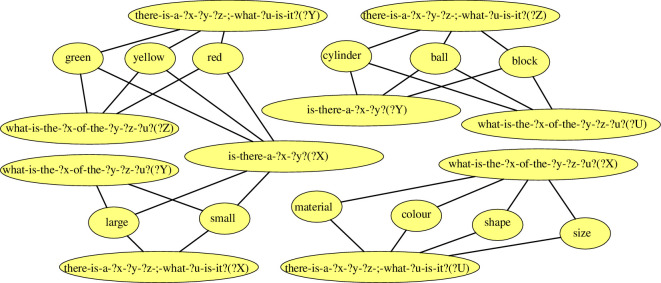
Fragment of a categorial network emerging from the experiment. The network captures how the arguments of constructions can fill the slots of other constructions. The entrenchment scores of the categorial links are not shown.

Along with an inventory of holophrase, item-based and lexical constructions, a network of grammatical categories emerges from the experiment. This network captures the association strength between constructions in terms of slots and their observed fillers. The nodes and links in the network are incrementally added by the application of repairs, while the entrenchment scores of the categorial links are updated after each observation. The categorial network at the end of the experiment counts 339.6 nodes and 1419.8 links on average. A fragment of such a network, with the entrenchment scores omitted, is shown in figure 14. In this figure, we see the categories of 12 arguments of 12 lexical constructions and their associations with the categories of 10 slots of 3 item-based constructions. For example, the category of the argument provided by the large-cxn, shown in the bottom-left part of the figure is compatible with the categories of three slots of three different item-based constructions. These are the ‘?Y’ slot of the what-is-the-?x-of-the-?y-?z-?u?-cxn, the ‘?X’ slot of the there-is-a-?x-?y-?z-;-what-?u-is-it?-cxn and the ‘?X’ slot of the is-there-a-?x-?y?-cxn. Alternative categories that are compatible with the ‘?X’ slot of this last construction are, according to the network, the categories of the arguments provided by the small-cxn, red-cxn, yellow-cxn and green-cxn. The grammatical categories of the emerged language, defined as generalizations that capture the behaviour of slots and their potential fillers, are captured by the combination of nodes and weighted links in the categorial network. This usage-based way of capturing the categories of a language is highly flexible, as it can elegantly include both frequent and rare categorial associations in a fine-grained manner. Based on the objectives and desires of the individual researcher, graph analysis techniques can straightforwardly be used to compute distances between nodes, or to cluster nodes into more coarse-grained categories. A complete visualization of this categorial network can be found in appendix B.

We finally conduct a secondary experiment, which consists in processing all observations from the test set using the grammars resulting from the different experimental runs of the primary experiment. The average communicative success amounts to a perfect 100% in both the comprehension and production direction. The average grammar size amounts to 101.5 constructions, of which 10.2 are holophrastic constructions, 57.1 are item-based constructions and 34.2 are lexical constructions.

## 6. Discussion and conclusion

The scientific contribution of the methodology and experiments presented in this paper is threefold. First of all, they provide computational evidence for the cognitive plausibility of constructivist theories of language acquisition. These theories attribute the ability of children to acquire language to two main cognitive capacities: intention reading and pattern finding. Intention reading deals with reconstructing the intended meaning of observed utterances, while pattern finding implements generalization processes that distil abstract schemata embodying the linguistic knowledge of a language user from these reconstructed utterance–meaning pairs. These schemata can then be used to fulfil the communicative function of language through the comprehension and production of natural language expressions. The methodology introduced in this paper presents a mechanistic model of the pattern-finding capacity. Based on utterances paired with a representation of their meaning, the learning algorithm gradually builds up an inventory of concrete to abstract form-meaning mappings, called constructions, along with a network of emergent grammatical categories that capture how the constructions of the grammar can combine to collaboratively comprehend and produce utterances. The experiments show that a meta-layer consisting of a small number of general repairs, which become active if an utterance cannot be successfully processed by the grammar learned so far, effectively leads to learning dynamics that are similar to those described in the psycholinguistic literature [1,14,45]. In the first phase of the learning process, the learner acquires holistic mappings between utterances and their meaning representation. Soon after that, holophrastic constructions are generalized to item-based constructions that integrate a variable slot. At the same time, this generalization process leads to the emergence of slot-filling constructions, here called lexical constructions. Along with the item-based and lexical constructions, a network of grammatical categories emerges, capturing the distribution of construction slots and their observed fillers. In a third phase, more abstract item-based constructions emerge, with an increasingly large number of variable slots. In the final phase of the learning process, most constructions have already been acquired and most remaining problems can be solved by adding new links to the categorial network. The learning dynamics are influenced by the degree of entrenchment of constructions and categorial links. Constructions that are often successfully used become more entrenched, while their competitors are suppressed. As a result of this process of entrenchment, the grammar reaches a stable state, while it remains adaptive to any changes in the discourse or environment. Similar dynamics have been observed in earlier experiments in the field of evolutionary linguistics, as for instance in the experiment on the emergence of compositionality in a population of autonomous agents by De Beule and Bergen [102].

The second contribution of the methodology and experiments presented in this paper concerns the corroboration of the theoretical underpinnings of construction grammar theories [3,4,10]. In particular, we provide a fully operational model of how a communicatively adequate linguistic system can be captured in the form of a collection of learned form-meaning mappings. These mappings can cover syntactico-semantic patterns of variable extent and degree of abstraction. The emerged constructions provide a unique insight into the compositional and non-compositional aspects of the learned language, as a consequence of the pattern-finding processes implemented by the repair strategies. Through these pattern finding processes, non-compositional pairings between aspects of observed form and meaning are included in constructions, while compositional aspects are generalized over through the use of variable slots. Like constructions, grammatical categories also emerge during the language acquisition process. In the spirit of radical construction grammar [4], categories are construction-specific and functionally motivated. They are conceived as fine-grained abstractions over observed syntactico-semantic usage patterns, and are captured in the form of a dynamic and adaptive categorial network.

Finally, the methodology and experiments presented in this paper pave the way for learning computationally tractable, large-scale, usage-based grammars that facilitate both language comprehension and production. The proposed learning algorithm supports online, interactive, incremental, transparent and data-efficient learning. The learner builds up its human-interpretable inventory of constructions and categories through the application of transparent syntactico-semantic generalization processes. Already after a single observation, the fragment of linguistic knowledge acquired by the learner can be successfully used for language comprehension and production. As more and more utterance–meaning pairs are observed, the linguistic knowledge of the learner quickly expands and becomes better fit for achieving their communication goals. As a result of the dynamics of rewarding successful construction applications and punishing competing ones, the grammar of the learner remains ever-adaptive to any changes in the task or environment. Owing to their online, interactive, incremental, transparent and data-efficient nature, the proposed mechanisms for learning computational construction grammars that facilitate both language comprehension and production can serve as an excellent basis for implementing the language acquisition ability of autonomous agents. At the same time, important challenges, limitations and scaffolds remain on different levels: (i) the model focuses on pattern finding only, scaffolding the intention reading process, (ii) the data to which the methodology is applied is still synthetic and thereby does not reflect actual language use and (iii) the model integrates repair strategies for meta-level learning during language comprehension only, limiting creative language use in the production direction.

## Figures and Tables

**Figure 15.**
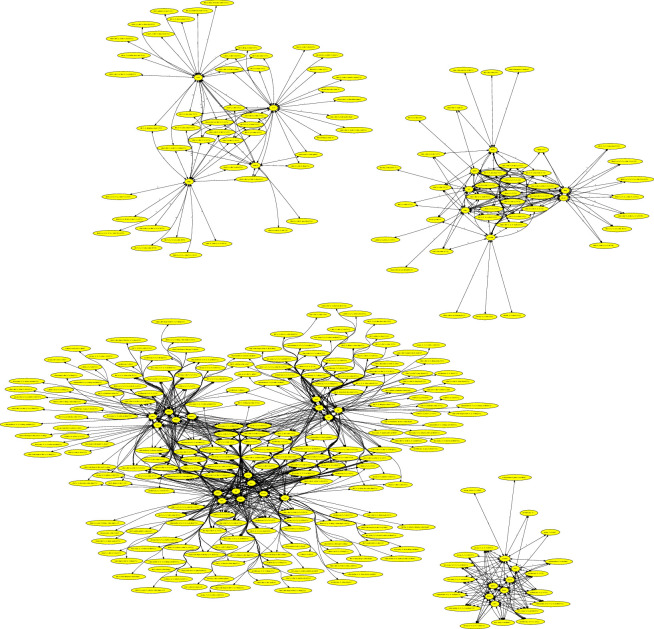
A categorial network after 2000 observations. We observe the formation of four separate connected components with clearly distinguishable hubs reflecting the learned grammatical categories. The first component, visualized here in the top-left corner, hosts all property types (e.g. ‘colour’, ‘material’, ‘size’). The second component, shown in the bottom-left corner, contains object properties (e.g. ‘green’, ‘metallic’, ‘small’). The third and fourth components, depicted in the top-right and bottom-right corners, incorporate the objects themselves, in their singular form (e.g. ‘object’, ‘cylinder’,‘ball’) and plural forms (e.g. ‘objects’, ‘cylinders’, ‘balls’), respectively. The numbers on the network edges reflect the frequencies with which slot-filler links have been observed by the learning agent.

**Figure 16.**
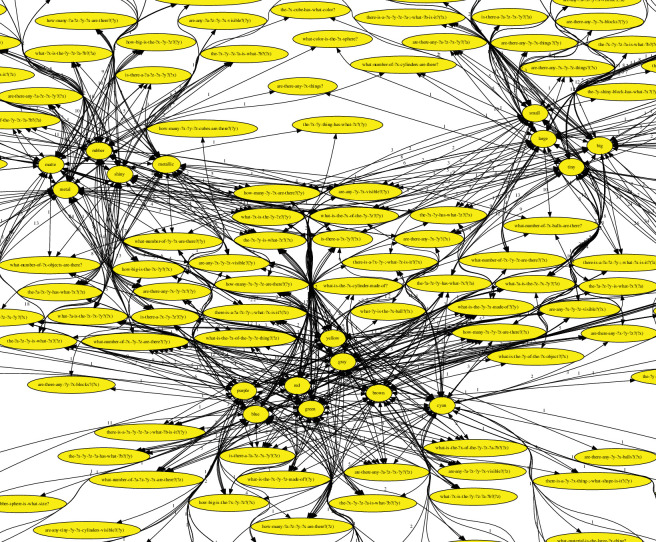
When zooming in on the object property component from the categorial network shown in figure 15, the network hubs become more clearly visible, with a cluster of colour terms in the middle of the bottom part of the figure standing out. The presence of such a cluster indicates that these nodes have frequently been observed in the same item-based slots. Nodes are clustered in terms of shared common neighbours by graphviz’ FDP renderer, using the Fruchterman–Reingold heuristic.

## Data Availability

Data available at [103].

## Appendix B

### B.1.Categorial network visualizations

Figures 15 and 16 show the agent's categorial network after 2000 observations.

^1^
We refer the interested reader to Refs [67] and [78] for examples of proof-of-concept operationalizations of intention reading.
